# Feasibility of surgical randomised controlled trials with a placebo arm: a systematic review

**DOI:** 10.1136/bmjopen-2015-010194

**Published:** 2016-03-15

**Authors:** Karolina Wartolowska, Gary S Collins, Sally Hopewell, Andrew Judge, Benjamin J F Dean, Ines Rombach, David J Beard, Andrew J Carr

**Affiliations:** 1 Oxford NIHR Musculoskeletal Biomedical Research Unit, Oxford, UK; 2 Nuffield Department of Orthopaedics, Rheumatology and Musculoskeletal Sciences, Botnar Institute of Musculoskeletal Sciences, University of Oxford, Oxford, UK; 3 Centre for Statistics in Medicine, Oxford, UK; 4 MRC Lifecourse Epidemiology Unit, University of Southampton, Southampton General Hospital, Southampton, UK; 5 Royal College of Surgeons of England Clinical Trials Unit, Botnar Institute of Musculoskeletal Sciences, Oxford, UK

**Keywords:** SURGERY, Randomised Controlled Trials, Placebos

## Abstract

**Objectives:**

To find evidence, either corroborating or refuting, for many persisting beliefs regarding the feasibility of carrying out surgical randomised controlled trials with a placebo arm, with emphasis on the challenges related to recruitment, funding, anaesthesia or blinding.

**Design:**

Systematic review.

**Data sources and study selection:**

The analysis involved studies published between 1959 and 2014 that were identified during an earlier systematic review of benefits and harms of placebo-controlled surgical trials published in 2014.

**Results:**

63 trials were included in the review. The main problem reported in many trials was a very slow recruitment rate, mainly due to the difficulty in finding eligible patients. Existing placebo trials were funded equally often from commercial and non-commercial sources. General anaesthesia or sedation was used in 41% of studies. Among the reviewed trials, 81% were double-blinded, and 19% were single-blinded. Across the reviewed trials, 96% (range 50–100%) of randomised patients completed the study. The withdrawal rate during the study was similar in the surgical and in the placebo groups.

**Conclusions:**

This review demonstrated that placebo-controlled surgical trials are feasible, at least for procedures with a lower level of invasiveness, but also that recruitment is difficult. Many of the presumed challenges to undertaking such trials, for example, funding, anaesthesia or blinding of patients and assessors, were not reported as obstacles to completion in any of the reviewed trials.

**Strengths and limitations of this study**

- Review of all published surgical randomised controlled trials (RCTs) with a placebo arm, spanning the years 1959 to 2014.
- Owing to the nature of this review, we could not investigate the obstacles that prevented initiation or completion of trials and, subsequently, our observations are limited to the successfully published trials. However, this review of all published trials provides different evidence than a report from a single discontinued trial.
- Many of the problems reported in reviewed trials are not unique to placebo-controlled surgical trials, but are also relevant to other surgical trials and randomised controlled trials in general.

## Introduction

Progress in surgery is based on practical experience.^1^ Surgical randomised controlled trials (RCTs) are uncommon;^2^ only about 15% of published RCTs are related to surgical interventions.^3^ Novel procedures tend to be developed through an iterative process of trial and error,^4^ and only 24% of the currently used surgical therapies are supported by results of RCTs.^1^

Apart from not being necessary for approval of new treatment,^5^ several reasons have been mentioned in the literature that may explain why surgical RCTs are scarce. Such studies are perceived as expensive^2^ ^6^ and unlikely to attract funding.^3^ ^5^ ^7^ They are considered to be difficult to design and conduct because of challenges posed by randomisation, blinding, differences in skills and experience of surgeons, variability of patients, as well as lack of consensus on surgical outcomes.^1^ ^2^ ^6–8^ Moreover, patient recruitment is also believed to be a problem.^6^ The inclusion of a placebo control adds another level of complexity to a RCT.^6^ ^9^ For example, some authors suggest that many patients may be unwilling to undergo an invasive procedure if there is no clear direct benefit to them, which may result in slow recruitment.^6^ Others believe blinding of patients and outcome assessors is not feasible, and that the surgeon can never be blinded.^10^ As a result of that, very few interventional procedures have been validated using a placebo-controlled RCT.^1^ ^2^ ^5^ ^9^ ^11^ ^12^ It is important to note that some of these opinions come from personal experience from a single trial, while others are just perceptions and assumptions. There have also been many publications discussing placebo in surgery that concentrate on ethical concerns, such as general equipoise and minimising the risks,^13^ ^14^ and on conceptual problems, for example, whether surgeons will be willing to test efficacy of an already established procedure.^10^ ^15^ However, very little has been written on the methodological challenges of such studies^6^ ^16^ and, to the best of our knowledge, no one has attempted to summarise the evidence from all the published placebo-controlled surgical trials.

When we previously performed a systematic review examining the harms and benefits of placebo-controlled surgical RCTs, we found that there clearly are obstacles to completing such trials, as less than a hundred have been published between 1959 and 2013.^12^ Therefore, we conducted a secondary review of these studies to find evidence corroborating or refuting persisting beliefs regarding the feasibility of carrying out placebo-controlled surgical trials.

## Methods

### Selection criteria

The criteria used to select placebo-controlled surgical RCTs have been described previously.^12^ In brief, studies were eligible if they were randomised trials in which the efficacy of surgery was compared with placebo. Surgery was defined as any interventional procedure that changes the anatomy and requires a skin incision or the use of endoscopic techniques; dental studies were excluded. We used the term ‘placebo’ to refer to a surgical placebo, a sham surgery, or a procedure intended to mimic the active intervention. A quasi-placebo, that is, a diagnostic procedure that could imitate the surgery, was also included. The important criterion was that patients were under general anaesthesia or blinded in some other way, and could not distinguish whether they underwent the actual surgery or placebo. We did not limit the inclusion criteria to any particular condition, patient group, intervention, or type of outcome. We excluded studies investigating anaesthesia or other pharmacological substances used perioperatively.

In this review, we used the term ‘surgical placebo’. The word ‘sham’ is preferred by some authors because surgical placebo has to involve an imitation of the investigated intervention in order to resemble it closely; therefore, it is different from an inactive ‘sugar pill’ placebo used in pharmacological trials.^17^ The word ‘sham’ has negative associations, and it suggests that a procedure is fake and deceitful; however, in many trials, the placebo involved an accepted surgical procedure such as endoscopy or arthroscopy, which was used also for diagnostic purposes with real benefits to the patients.

### Search strategy

We searched the MEDLINE, EMBASE and the Cochrane Central Register of Controlled Trials databases from the date of their inception to 14 November 2013, with no restriction on language. We did not systematically search for studies reported only as conference abstracts. Search terms were published previously.^12^

Three reviewers (KW, IR and BJFD) independently screened the initial set of records identified from the search, and then screened the full text of any potentially relevant articles. Each reviewer assessed the eligibility of each study, and the final list of included studies was agreed by consensus. Moreover, we searched ClinicalTrials.gov (on 14 November 2013), a database of registered randomised clinical trials, to identify any recently completed or ongoing studies. On 15 June 2014, we checked whether results of any of the trials identified in the ClinicalTrials.gov database have been published since the original search.

### Dealing with duplicate publications

When there were several articles reporting outcomes from a single trial, that is, with the same authors, location, patient population and recruitment dates, we only included the paper reporting the primary outcome for the trial, and excluded pilot and follow-up reports.

### Data extraction

We used a standardised data extraction form to collect information about the characteristics of each study including: year of publication, country in which the trial was conducted, funding source, details of the active and placebo intervention as well as the type of anaesthesia, blinding, number of patients who were assessed, eligible, randomised and who declined participation, as well as those who completed the trial. To reduce errors, the three review authors (KW, IR and BJFD) extracted data separately and checked the entries for consistency; a single set of data was agreed by all three reviewers.

### Data synthesis

We have performed a descriptive analysis of the characteristics of each individual study and presented data in a table.

## Results

### Study selection

**Figure 1:**
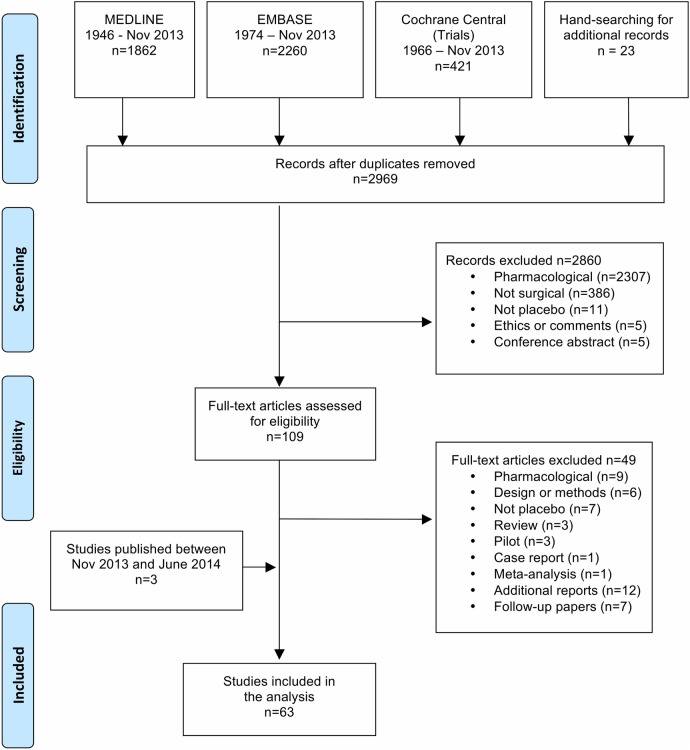
PRISMA flow diagram.

We analysed the studies identified as a part of the systematic review on harms and benefits, including seven trials that were excluded from the systematic review due to lack of a direct comparison between the surgical and the placebo group. We also checked whether the trials identified in the ClinicalTrials.gov database had their results published between November 2013 and June 2014, and found three additional trials.^18–20^ This resulted in 63 full-text articles, which were included in this review (figure 1).

### Placebo-controlled surgical RCT characteristics

The number of published placebo-controlled surgical trials was small; however, 73% (n=46/63) of included RCTs were published after the year 2000, suggesting an increasing interest in performing such studies. Half the trials (n=35/63, 55%) used a key-hole surgery, including endoscopy (n=28/63), laparoscopy (n=4/63), arthroscopy (n=2/63) and bronchoscopy (n=1/63). The remaining trials involved other types of minimally invasive interventions, for example, using catheters for vascular access, or needles for injection of fat or exogenous materials to remodel tissue. Very few studies investigated open techniques, such as exposure of the internal mammary artery (n=2/63), or exposure of scalp muscles (n=1/63). Fifteen trials used implants and an additional seven used gastric balloons or bubbles (characteristics of the reviewed trials are presented in online supplementary appendix 1).

10.1136/bmjopen-2015-010194.supp1 Supplementary appendix

### Funding sources

One-third of the studies (n=21/63, 33%) were non-commercially funded, and almost as many were funded by a commercial company (n=18/63, 29%), often the manufacturer of the implant or the endoscope. The source of funding in the remaining studies (n=24/63, 38%) was not reported.

Over half the trials were undertaken in the USA (n=35/63, 56%), the others were in Canada, the UK, Germany, The Netherlands, Belgium, Italy, Finland, Sweden, Portugal, Norway, Greece, Denmark, Australia and Brazil.

### Sample size

The majority (n=47/63, 75%) of the identified studies were small, with fewer than 100 participants. The median number of patients randomised in a trial was 61 (IQR 66, range 10–298).

About half the RCTs (n=33/63; 52%) reported a formal sample size calculation, but only a quarter (n=16/63) allowed for dropouts and attrition. Most of the trials that included a sample size calculation (n=23/33, 70%) attained their prespecified sample size (without accounting for attrition). Ten trials under-recruited, such that the number of randomised patients was lower than the calculated sample size. All 10 trials were terminated early: three due to slow recruitment,^21–23^ one because at the interim analysis the surgery was highly effective,^24^ and two because at the interim analysis the active procedure lacked efficacy.^18^ ^25^ Two studies were stopped because of serious adverse events either in the trial^26^ or at another centre using a similar procedure.^27^ One trial was terminated when the sponsoring company was sold.^28^ Finally, one study was stopped because the investigated procedure was approved as a standard care and the equipoise ceased to exist, despite the fact that the study did not show its superiority over placebo.^29^ Finally, one trial recruited the intended sample size, but due to a high drop-out rate the number of patients who completed the trial was lower than the required sample size.^30^

### Recruitment and screening

Recruitment, sometimes as slow as 1–2 patients per month,^21^ ^31^ was a common problem,^18^ ^21^ ^27–35^ and was the reason for an early termination of three trials.^21–23^

Many of the analysed studies did not provide any details about screening and recruitment; they either stated that they recruited consecutive patients fulfilling the criteria,^21^ or that they randomised patients who were willing to participate and were eligible.^36^ About one-third of the trials specified the number of screened (n=24/63, 38%) and eligible patients (n=27/63, 43%), and stated how many patients declined participation or withdrew before the treatment (n=22/63, 34%); only one-fifth of the trials (n=13/63, 21%) reported all three numbers (see online supplementary appendix 1). The available data suggest that the initial assessment of eligibility was the main obstacle in recruitment as patients did not meet the inclusion criteria or were not eligible due to exclusion criteria.

On average, it was necessary to screen more than five patients in order to randomise one, but three in four eligible patients started the trial (table 1). The number of patients who had to be screened before the necessary group was recruited varied greatly. This variance was, at least partly, related to the method of identifying potential participants. The trial with the largest number of screened patients recruited participants by using TV and newspaper advertising: out of 4523 screened patients only 260 were eligible and were willing to participate; however, 196 had negative discography, and only 64 patients were randomised.^23^ More targeted recruitment from specialist centres had much higher success rates, but often required a multisite effort.^35^

**Table 1:** Participants flow through the reviewed trials as a percentage of the number of patients who were randomised into each trial

	Number of studies	Median per cent of sample randomised	First and third quartile (%)	Minimum and maximum (%)
Screened	24	530	243, 773	100, 7067
Eligible	27	132	108, 172	100, 448
Declined	22	18	8, 144	3, 4942
Sample size	33	96	90, 110	49, 323
Outcome assessed	61	96	90, 100	52, 100

‘Number of studies’ refers to the number of trials that provided relevant data. Trials terminated early are included in these analyses. ‘Sample size’ refers to the sample size required to reach statistical power, not inflated to account for drop-outs. ‘Outcome assessed’ refers to total number of patients, in both arms. The denominator is the number of patients actually randomised into each trial.

Many trials had additional inclusion and exclusion criteria that could only be verified after the patient entered the trial, for example, a verification of diagnosis by positive findings during the endoscopy, or on diagnostic imaging. As a consequence of this, many patients were excluded because either they did not have the investigated condition or they had some concomitant condition that precluded their participation in the trial and, sometimes, required appropriate treatment. Moreover, any technical complications during the assessment or study procedures potentially resulted in patients’ drop-out. For example, in the trial on laparoscopic adhesiolysis for abdominal pain by Swank and colleagues,^32^ nine patients did not have adhesions, one of them had a hernia and was treated laparoscopically, three patients had stricturing adhesions that required therapeutic adhesiolysis, and in one instance a pneumoperitoneum could not be achieved; therefore, out of 121 assessed patients, 13 were excluded during laparoscopy.

Fluctuating symptoms were a problem in a few studies, for example, patients became asymptomatic while waiting for the procedure and had to be excluded from the trial,^19^ or did not report symptoms during the study visit, and did not undergo the treatment, but were included in the intention-to-treat analysis.^37^ This problem also complicated the post-treatment assessment,^38^ especially that only one trial included an observational control group.^39^

### Refusal to participate

Some of the approached patients declined participation in the trial, withdrew their initial consent, refused to be randomised or to comply with the requirements of the protocol, and had a strong preference for one of the treatment options. Most of the trials did not report the reasons for patients’ refusal to participate, and the available data did not allow us to quantify the percentage of patients who refused to enter the study. Only 22 reviewed trials stated the number of patients who declined to participate, but it was not always clear whether these numbers referred to patients at the screening stage or to patients already identified as eligible. The median percentage of patients who declined participation as a percentage of randomised patients was 18%, and varied from 3% to 4842%. It is important to note, that the two trials with high numbers of patients refusing to participate investigated vertebroplasty, which, at the time, was an established procedure; therefore, patients could easily receive the treatment from a different medical centre, without participating in a trial.^34^ ^35^

### Patient retention

In general, recruitment was more problematic than retention and, once recruited, patients usually remained in the trial. Across the reviewed trials, 96% of randomised patients completed the study (table 1). A lower completion rate in five trials was caused by an early termination^25^ ^29^ ^40^ as well as withdrawals or change of patients’ health status.^41^ ^42^ In general, the predicted attrition, by which the required sample size was inflated to account for drop-outs, was 10% (median) with the range from 5% to 24%, whereas the actual patients’ attrition between randomisation and outcome assessment was 4% (range 0–50%).

The completion rate was similar in the active and in the placebo arm, except for two trials: one^18^ where five times as many patients were lost to follow-up in the active group than in the placebo group, and one^30^ where the drop-out rate was three times higher in the placebo group. Neither of these studies could explain this difference.

Most of the drop-outs occurred before randomisation. The reported reasons for drop-out during the trial were withdrawals, loss to follow-up, or discontinuation without known cause,^20^ ^39^ ^41^ ^43^ ^44^ patients’ request to be unblinded,^24^ adverse events,^25^ ^26^ change of medical status such as pregnancy or concurrent illness.^23^ ^45^ ^46^ A long wait between the screening and procedure did not necessary result in patient withdrawal.^43^ A variable reporting did not allow us to evaluate quantitatively the reasons for drop-outs.

### Blinding was possible, and some studies attempted to blind surgeons

In 12 trials (19%), only patients were blinded, but in the majority of RCTs (n=51/63, 81%) both patients and outcome assessors were blinded; including three trials, in which there was also an attempt to blind the operator. For example, in two trials, the implant delivery system was preloaded by the manufacturer—the devices looked identical but only one contained an implant.^28^ ^47^ In another trial, the surgeon placed the catheter but then handed the procedure over to a technician who delivered the treatment according to the randomisation.^21^

Authors of the reviewed trials went to great lengths to imitate the visual, verbal and physical cues, and to make the placebo as similar as possible to the active procedure. For example, patients wore goggles, or had the view obscured, so that they could not see the device.^48^ The preparation for the placebo intervention was done in the same way as for the active procedure.^35^ ^49^ Similar verbal instructions were given as during the surgery,^42^ ^50^ and there were attempts to imitate the noises made by the devices.^51^ In trials that used exogenous substances, the container was opened so that the distinct smell was also present during the placebo condition.^35^ Some researchers attempted to keep the duration of the procedure the same in both arms,^39^ ^43^ ^52^ whereas others thought that it was more ethical to shorten the placebo intervention.^31^

Very few studies assessed the success of blinding. Often authors thought that it was reasonable to assume that patients in the study were not able to distinguish between placebo and surgery due to minimally invasive characteristics of the procedure, and minimally postoperative treatment-related symptoms.^53^ ^54^ In one trial, the post-treatment symptoms were believed to be a sign of correctly placed effective gastroplication, as patients with these symptoms had better outcomes.^39^ Blinding was reported as successful in n=13/63 (21%) studies. In four trials,^35^ ^42^ ^55^ ^56^ a larger proportion of patients in the active group guessed correctly; however, the placebo group did not guess the treatment allocation. In one study, two patients were definitely unblinded early due to implant extrusion.^57^

### Anaesthesia

In the reviewed trials, patients in both groups received some type of anaesthesia. General anaesthesia or sedation was used in n=26/63 trials (41%), including one trial in which general anaesthesia was used in the surgical group, but patients in the placebo group were sedated without intubation.^29^ Local analgesia was used in n=16/63 (25%) RCTs, four studies used a mixture of methods, and n=17/63 (27%) trials did not describe the type of anaesthesia used. None of the trials reported that anaesthesia was a barrier in conducting their study.

## Discussion

This review has demonstrated that surgical RCTs with a placebo arm are feasible, at least for procedures with a lower level of invasiveness. Many of the presumed challenges, such as funding, anaesthesia or blinding of patients and assessors, were not reported as obstacles in any of the reviewed trials. The main hurdle in completing a trial was finding a sufficient number of eligible patients.

We found that, although, there were very few surgical RCTs with a placebo arm published between 1959 and 2014, there was a rising trend. This may be related to an increasing interest in placebo and placebo-controlled trials in general,^58^ or to the increasing popularity of minimally invasive procedures since 1980s. The latter explanation is supported by the fact that most of the reviewed trials used some type of key-hole surgery.

The analysed placebo-controlled trials were funded equally often by industry as by non-commercial funding bodies. The number of commercially funded older trials may be underestimated in our review because surgical RCTs funded by industry have lower odds of being published.^59^ However, the recent trials are registered in the ClinicalTrials.gov database and would have been identified. The distribution of the source of funding was similar to that described by other authors.^11^ This is encouraging, as it shows that there is an interest within the industry to validate the efficacy of their products, and also that the non-commercial bodies are willing to investigate the efficacy of surgical procedures. The costs of running surgical RCTs are high,^2^ but in the long run, preferential funding of treatment with proven efficacy may help to improve the allocation of resources and to lower the costs of healthcare.^60^ For example, the trial by Moseley and colleagues^52^ demonstrated that arthroscopic surgery had no benefits because the outcomes in the arthroscopic debridement arm and the lavage arm were not better than in the placebo group and, consequently, there was a decline in the use of this procedure for knee osteoarthritis.^61^

Recruitment into placebo-controlled surgical trials was possible but was often very slow, and resulted in an early termination of several trials. Slow recruitment is the most frequent reason for discontinuation of RCTs, including surgical RCTs. For example, 21% of reviewed surgical RCTs were discontinued early, and 44% of these were due to problems with recruitment.^59^ Authors often underappreciate the fact that the target population in surgical trials is small; therefore, it may be challenging to recruit a required number of patients in a reasonable period of time.^2^ The right timing of a trial may also affect its completion,^7^ for example, initiating a trial too early in the intervention's development may result in more procedure-related adverse events,^26^ whereas, when a procedure has been already established, like vertebroplasty, it may be difficult to recruit participants.^34^ ^35^

In the reviewed trials, the number of patients who had to be screened in order to recruit a necessary participant group was larger than in other RCTs, but the proportion of eligible patients who started the study was comparable with other types of RCTs.^62^ This is another argument suggesting that the main challenge in those trials was finding suitable patients rather than persuading potential participants to enter the trial, and this is a bigger problem than in other types of RCTs.^62^ Reporting of the recruitment process and eligibility was generally poor and often difficult to interpret, as the reviewed studies usually did not describe in detail why eligible patients did not enter the trial, which is in line with observations from other reviews.^62^ The quality of reporting in analysed RCTs was poor, but this is a known problem in surgical trials.^7^ ^62^

There is an assumption that patients are unwilling to take part in surgical RCTs, especially patients in severe pain.^63^ Interestingly, in the trial by Moseley and colleagues,^52^ patients in more pain were more likely to agree to participate. Also patients tend to choose the new treatment even if it was not proven to be superior over placebo. For example, in the trials on Parkinson's disease, patients actually opted for the transplantation when they were given a choice after the end of the trial, despite the fact that it was not demonstrated to be more effective than placebo.^49^ In a recent orthopaedic placebo-controlled RCT, patients were willing to participate, and screening failures were a larger problem than refusals or withdrawals.^16^ The clinical characteristics of patients who entered into a placebo-controlled RCT were comparable to the non-enrolment group, as well as to patients in other trials.^16^

Only about half the published trials reported a sample size calculation, which is in line with another review of surgical trials, which found that sample size calculations were reported only in 63% of RCTs.^11^ However, it is important to note that some of the reviewed trials were published before the CONSORT (Consolidated Standards of Reporting Trials) were introduced, and before the sample size calculation became required by the board review. Some trials were small because of the author's assumption that surgical studies have a large effect size; therefore, inferring that a smaller sample size is required in surgical trials than in drug trials.^47^ ^64^ However, surgical RCTs may require larger numbers of patients to reach the required sample size.^65^ Recent systematic reviews demonstrated that the effect size of the surgical procedure in comparison with placebo in the existing trials was often small.^12^ ^66^ It is likely that the apparent lack of difference between the active treatment and placebo might have been related to the small sample size and the effect not reaching the statistical significances.^47^ ^67^ ^68^ It might be also caused by a large placebo effect; however, the magnitude of the placebo effect in surgical procedures is unknown. The magnitude of response in the placebo arm is related not only to the placebo effect, that is, response directly related to the placebo intervention, but also to non-specific changes, such as regression to the mean, natural history of disease, or effect of participation in the trial.^69^ Only one reviewed trial included a non-interventional group to control for these non-specific effects.^39^

A placebo procedure can successfully imitate a minimally invasive surgery. Blinding in interventional trials is more challenging than in pharmaceutical ones;^11^ ^70^ however, there are many strategies to blind the patients and outcome assessors,^70^ and the reviewed trials often used ingenious methods to achieve blinding. The success of blinding was rarely assessed, but it is not necessary, according to the current reporting standards. The requirement to assess blinding was removed from the CONSORT checklist because of evidence that testing for blindness is not valid because it cannot distinguish the success of blinding from ‘hunches’ about the treatment's efficacy.^71^

Blinding of patients and outcome assessors is especially important if the outcomes are subjective or difficult to quantify.^72^ Softer outcomes are difficult to evaluate in unblinded trials due to patient-related or assessor-related bias, which may distort the treatment effect.^73^ ^74^ In this analysis, we have demonstrated that the withdrawal rate was generally low and was similar in the active and the placebo groups. This provides supporting evidence that blinding reduces the attrition bias, as patients do not know to which treatment they had been allocated.^75^

### Future implications for clinicians and unanswered questions

What remains to be understood is why eligible patients decline participation or withdraw their consent before randomisation.^76^ Addressing these issues may improve the recruitment procedure in future trials.

There is also a need to estimate the magnitude of placebo effect in interventional trials. Several authors have highlighted the fact^12^ ^65^ ^66^ that for softer outcome measures, the magnitude of placebo effect in surgical trials is underestimated while the effect size of the surgical intervention is overestimated and, as a result of that, many trials do not recruit sufficient numbers of patients to detect differences between the effects of surgery and placebo.

Journals should encourage authors to report the details of patient recruitment and allocation, including the reasons for withdrawals and screening failures. Data like this are very useful when planning future trials. There has been an improvement in the reporting quality of recent trials,^20^ and these guidelines were included in the CONSORT extension for non-pharmacological interventions.^77^

In conclusion, not every surgical procedure has a viable placebo control; however, surgical RCTs with a placebo arm are feasible for many less invasive procedures. Although placebo-controlled surgical RCTs are challenging, they should not be dismissed as a potential trial design in surgical research. There is a need to better understand the factors that make those trials challenging so that future trials are not terminated early, and contribute good quality evidence to surgical practice.
