# Supplementary material for: Feasibility of surgical randomised controlled trials with a placebo arm: a systematic review
Source: BMJ Open. 2016 Mar 15;6(3):e010194. doi: 10.1136/bmjopen-2015-010194 (PMC4800115; doi:10.1136/bmjopen-2015-010194)
Supplement: Supplementary appendix [file bmjopen-2015-010194supp_appendix.pdf]

Appendix 1 Characteristics of the reviewed trials

Study	Year	Condition	Active intervention	Placebo intervention	Country	Screened as % of randomised	Eligible as % of randomised	Declined as % of randomised	Number of randomised	Completed as % of randomised	Completed as % of randomised in surgery arm	Completed as % of randomised in placebo arm	Calculated sample size	Completed as % of sample size	Randomised as % of sample size	Blinding	Analgesia
Abbott et al.	2004	Endometriosis	Laparoscopy + ablation	Laparoscopy	UK	NA	323%	6%	52	75%	100%	100%	40^	98%	130%	P&A	NA
Arts et al.	2010	GERD	Endoscopy + RF treatment	Endoscopy + setup but no RF delivery	Belgium	NA	NA	NA	22	100%	100%	100%	22	100%	100%	P&A	SE (1subj GA)
Baeck et al.	2009	Sleep apnea	RF surgery of the palate	Applicator insertion but no RF delivery	Finland	250%	106%	6%	32	100%	100%	100%	26^	123%	123%	P	LA
Bajbouj et al.	2009	Globus sensation	Endoscopy + ablation using argon plasma coagulation	Endoscopy + connected applicator but no current	Germany	NA	NA	NA	21	90%	91%	90%	40	48%	53%	P&A	SE
Benjamin et al.	1988	Obesity	Endoscopy + gastric bubble + diet	Endoscopy + balloon imitation + diet	USA	NA	NA	NA	90	68%	crossover	crossover	NA	NA	NA	P&A	SE
Bradley et al.	2002	Osteoarthritis	Tidal irrigation of the joint	Saline injection and leg manipulation	USA	NA	NA	NA	180	99%	98%	100%	150^	119%	120%	P&A	LA
Buchbinder et al.	2009	Osteoporotic vertebral fractures	Percutaneous vertebroplasty	Injection of anaesthetic but not cement +cephalosporin	Australia	600%	281%	181%	78	94%	95%	93%	48	152%	163%	P&A	LA
Castro et al.	2010	Severe asthma	Bronchoscopy + RF treatment	Bronchoscopy + placebo procedure	USA, Brazil, Canada, Australia, UK	201%	103%	3%	288	97%	92%	96%	225	124%	128%	P&A	SE
Cobb et al.	1959	Coronary disease	Ligation of internal mammary artery	Skin incision and exposure of vessels but no ligation.	USA	NA	NA	NA	17	100%	100%	100%	NA	NA	NA	P	LA
Corley et al.	2003	GERD	Endoscopy + RF treatment	Endoscopy + setup but no RF delivery	USA	NA	NA	NA	64	81%	89%	72%	64	81%	100%	P&A	SE
Cotton et al.	2014	Sphincter of Oddi dysfunction	Endoscopy + sphincterectomy + ERCP	Endoscopy + ERCP	USA	740%	169%	35%	214	81%	84%	75%	193^	90%	111%	P&A	SE
Davys et al.	2005	Plantar callosities in RA	Scalpel debridement of the callosity	Simulation using blunt-edged scalpel	UK	145%	134%	34%	38	100%	100%	100%	38	100%	100%	P	NA
Deviere et al.	2005	GERD	Endoscopy + a nonresorbable copolymer	Endoscopy without implant + prophylactic antibiotics	Germany, Belgium, Italy	NA	NA	NA	64	100%	100%	100%	NA	NA	NA	P	SE
Dimond et al.	1960	Coronary disease	Ligation of internal mammary artery and vein	Skin incision and exposure of vessels but no ligation	USA	NA	NA	NA	18	100%	100%	100%	NA	NA	NA	P	LA
Dowson et al.	2008	Migraine	Implant for patent foramen ovale + heparin	Skin incision in the groin + transoesophageal US + aspirin and clopidogrel - no heparin	UK	301%	111%	NA	147	93%	88%	97%	132^	103%	111%	P&C&A	GA
Eid et al.	2014	Obesity	Endoscopy + gastroplication (StomaphyX)	Endoscopy	USA	848%	316%	206%	90	82%	76%	94%	135^	55%	67%	P	GA
Fleischer et al.	1985	Bleeding esophageal varices	Endoscopy + laser + cimetidine or antacids + vasopressin if bleeding persisted	Endoscopy + setup (laser was turned on, a verbal order was given to activate the laser but not used) + cimetidine or antacids after endoscopy + vasopressin if bleeding persisted	USA	NA	NA	NA	20	100%	100%	100%	NA	NA	NA	P&C&A	NA
Fockens et al.	2010	GERD	Endoscopy + Gatekeeper implant	Endoscopy + saline instead of implant and instead of antibiotics	USA , Netherlands	335%	150%	NA	118	65%	68%	60%	NA	NA	NA	P	SE
Freed et al.	2001	Parkinson's disease	Fetal dopamine neurons transplantation+trepanation + PET + MRI + phenytoin	Incomplete trepanation (dura intact) + PET + MRI + phenytoin - sham-transplantation	USA	NA	NA	NA	40	98%	95%	100%	NA	NA	NA	P	LA
Freeman et al.	2005	Discogenic low back pain	Electrothermal therapy	Catheter inserted but not connected + cephazolin +CT	Australia	NA	NA	NA	57	96%	95%	100%	75	73%	76%	P&S&A	SE
Freitas et al.	1985	Bleeding from gastric/duodenal ulcers	Endoscopy + electrocoagulation + cimetidine	Sham + cimetidine	Portugal	615%	215%	NA	78	100%	100%	100%	NA	subgroups	NA	P	SE
Friedman et al.	2008	Sleep apnea	Palatal implants	Identical implementation device without an implant + antibiotics	USA	181%	129%	NA	62	89%	94%	84%	54^	102%	115%	P&A	NA
Fullarton et al.	1989	Bleeding from peptic ulcers	Endoscopy + heater probe + ranitidine	Endoscopy + heater probe activated in the gut lumen + ranitidine	UK	1465%	119%	NA	43	100%	100%	100%	NA	NA	NA	P&C&A	NA
Geenen et al.	1989	Sphincter of Oddi dysfunction	Endoscopy + sphincterectomy + ERCP + manometry + morphine/neostigmine provocation test	Endoscopy + device activated in the lumen of the duodenum + ERCP + manometry + morphine/neostigmine provocation test	USA	615%	109%	9%	47	100%	100%	100%	NA	NA	NA	P&C&A	NA

Geliebter et al.	1990	Obesity	Endoscopy + balloon	Endoscopy + deflated balloon	USA	NA	NA	NA	10	100%	100%	100%	NA	NA	NA	P&A	LA
Genco et al.	2006	Obesity	Endoscopy + balloon (BioEnterics)	Endoscopy but no balloon	Italy	NA	NA	NA	32	100%	100%	100%	NA	NA	NA	P&A	SE
Gillespie et al.	2010	Sleep apnea	Palatal implants	Identical implementation device without an implant	USA	NA	NA	NA	51	98%	NA	NA	80^	63%	64%	P&S&An	LA
Gross et al.	2011	Parkinson's disease	Pigmental cells transplantation	Scalp incisions and partial-thickness burr holes + MRI - the same duration	USA, Germany	221%	135%	10%	71	94%	89%	100%	68	99%	104%	P&A	GA
Guyuron et al.	2009	Migraine	Surgical "deactivation" of migraine trigger points	Exposure of muscles and nerves without changing their integrity	USA	417%	100%	NA	76	99%	100%	100%	NA	NA	NA	P&A&An	NA - occipital group GA, other - not stated
Hartigan et al.	1994	Esophageal varices	Endoscopy + sclerotherapy	Endoscopy + placebo solution released to the gut lumen	USA	NA	116%	16%	253	100%	100%	100%	244^	104%	104%	P	NA
Hogan et al.	1989	Obesity	Endoscopy + gastric bubble	Endoscopy + sham insertion	USA	271%	NA	NA	59	95%	100%	88%	NA	NA	NA	P&A	SE
Jarrell et al.	2005	Endometriosis	Laparoscopy + biopsy + sharp excision	Laparoscopy + biopsy	Canada	NA	NA	3%	29	52%	47%	57%	84^	18%	35%	P&C&A	NA
Kallmes et al.	2009	Osteoporotic vertebral fractures	Percutaneous vertebroplasty	Simulated (audio, sensory, even smell) vertebroplasty - injection of anaesthetic but not cement	USA, UK, Australia	1384%	329%	229%	131	98%	99%	97%	130	98%	101%	P&A	LA
Koutsourelakis et al.	2008	Sleep apnea	Septoplasty	Simulated resection with manipulation of instruments - the same amount of time	Greece	NA	104%	4%	49	100%	100%	100%	24	204%	204%	P&A	LA
Laine et al.	1987	Upper GI tract bleeding (ulcers)	Endoscopy + electrocoagulation	Endoscopy + probe activated in the lumen of the gut	USA	748%	NA	16%	44	93%	100%	100%	NA	NA	NA	P&C&A	NA
Lee et al.	2001	Urinary stress incontinence	Autologous fat injection	Fat harvested but discarded + saline injection + trimetoprim-sulfamethoxazole or nitrofurantoin	Canada	NA	NA	NA	68	82%	77%	88%	90	62%	76%	P&A	LA and SE occasional GA
Leon et al.	2005	Coronary disease	Percutaneous myocardial laser revascularisation	Setup but no laser procedure	USA	NA	NA	NA	298	100%	100%	100%	NA	NA	NA	P&A	NA
Lindor et al.	1987	Obesity	Endoscopy + balloon + diet	Endoscopy + empty introducer tube + diet	USA	NA	NA	NA	22	95%	91%	100%	71	30%	31%	P&A	SE
MacLeod et al.	1983	Bleeding from a peptic ulcers	Endoscopy + laser + cimetidine	Endoscopy + cimetidine	UK	1551%	120%	NA	45	100%	100%	100%	NA	NA	NA	P	NA
Mathus-Vliegen et al.	1990	Obesity	Endoscopy + balloon	Endoscopy + manipulation without balloon insertion + simulated "click" of device disconnection	Netherlands	NA	NA	NA	28	96%	100%	100%	NA	NA	NA	P&C&A	SE
Maurer et al.	2012	Sleep apnea	Palatal implants	Identical implementation device without an implant	Germany	NA	NA	NA	22	91%	91%	91%	NA	NA	NA	P&C&A	LA
Meshkinpour et al.	1988	Obesity	Endoscopy + balloon	Endoscopy + empty introducer tube + simulation of inflation process	USA	NA	265%	NA	23	91%	100%	100%	NA	NA	NA	P&A	SE
Montgomery et al.	2006	GERD	Endoscopy + EndoCinch plication technique	Endoscopy - the same duration	Sweden	NA	NA	NA	46	93%	100%	88%	NA	NA	NA	P&C&A	GA
Moseley et al.	2002	Osteoarthritis	Arthroscopy + debridement with chondroplasty but not spur removal or arthroscopy + lavage	Skin incision without arthroscopy	USA	NA	180%	80%	180	91%	90%	92%	164^	99%	110%	P&A	GA but SE and no intubation in placebo
Olanow et al.	2003	Parkinson's disease	Fetal tissue transplantation + antibiotics + cyclosporine + PET	Partial burr holes + antibiotics + cyclosporine + PET	USA	NA	NA	NA	34	91%	NA	NA	NA	NA	NA	P&A	GA
Pauza et al.	2004	Discogenic low back pain	Electrothermal therapy +discography + CT + prophylactic antibiotics + analgesics + rehabilitation	Introducing a needle onto the disc (visual and auditory feedback)+discography + CT + prophylactic antibiotics + analgesics + rehabilitation.	USA	7067%	NA	4942%	64	88%	86%	89%	67	84%	96%	P&C&A	LA
Porter et al.	2006	Turbinate hypertrophy	RF surgery	Placement of the probe, anaesthesia, and sound from the RF generator	USA	NA	NA	NA	32	100%	100%	100%	NA	NA	NA	P	LA
Rothstein et al.	2007	GERD	Endoscopy + plication	Endoscopy + setup but device not activated	USA, Germany, Belgium	NA	NA	NA	159	82%	81%	85%	NA	NA	NA	P&A	SE

Salem et al.	2004	Coronary disease	Percutaneous myocardial laser revascularisation	Setup but no laser activated	Norway	NA	NA	NA	82	96%	98%	95%	78^	101%	105%	P&A	NA
Schwartz et al.	2007	GERD	Endoscopy + gastroplication (Endocinch)	Endoscopy + setup without needle and thread loaded	Netherlands	NA	NA	NA	60	95%	100%	100%	54^	106%	111%	P&A	SE
Scolapio et al.	2001	Dysphagia	Endoscopy + balloon cathether (temporary inflation)	Endoscopy + balloon cathether - not inflated	USA	NA	NA	NA	86	NA	NA	NA	NA	NA	NA	P	SE
Shaheen et al.	2009	Barrett's oesophagus	Endoscopy + RF ablation+ biopsy + esmoprazole	Endoscopy + biopsy + esmoprazole	USA	594%	150%	158%	127	92%	93%	91%	NA	NA	NA	P&An	SE
Sihvonen et al.	2013	Degenerative meniscus tear	Arthroscopic partial meniscectomy	Arthroscopy and sham	Finland	NA	140%	16%	146	100%	100%	100%	112^	130%	130%	P&C&A	NA
Silverberg et al.	2008	Alzheimer's disease	Ventriculoperitoneal shunt + brain irrigation + ventricular fluid exchange	Identical shunt but occluded	USA	171%	120%	20%	230	71%	80%	63%	256	64%	90%	P&C&A	GA
Steward et al.	2008	Sleep apnea	Palatal implants	Identical implementation device without an implant + antibiotics	USA	968%	448%	348%	100	100%	100%	100%	100	100%	100%	P&C&A	LA
Stone et al.	2002	Coronary disease	Percutaneous coronary intervention + percutaneous myocardial laser revascularisation	No placebo intervention but patients were blinded during the percutaneous coronary intervention	USA	NA	NA	NA	141	100%	100%	100%	128^	110%	110%	P&C&A	SE
Stuck et al	2005	Snoring	RF surgery of the palate	Device was inserted but not activated	Germany	NA	NA	NA	26	88%	92%	85%	24^	96%	108%	P&A	LA
Sutton et al.	1994	Endometriosis	Laparoscopy + laser ablation + adhesiolysis + uterine nerve ablation	Laparoscopy	UK	NA	100%	NA	74	85%	NA	NA	NA	NA	NA	P&A	NA
Swank et al.	2003	Chronic abdominal pain	Laparoscopy + adhesiolysis	Laparoscopy	Netherlands	NA	NA	8%	100	96%	98%	94%	100	96%	100%	P&A	NA
Thompson et al.	2013	Obesity	Endoscopic Suturing for Transoral Outlet Reduction	Sham	USA	465%	168%	100%	77	90%	NA	NA	132	52%	58%	P&A	GA
Thomsen et al.	1981	Meniere's disease	Endolymphatic sac decompression	Simple mastoidectomy	Denmark	100%	NA	NA	30	100%	100%	100%	NA	NA	NA	P&A	NA
Toouli et al.	2000	Sphincter of Oddi dysfunction	Endoscopy + sphincterectomy + ERCP + manometry + morphine/neostigmine provocation test	Endoscopy + papillotome introduced into duodenum, noise made but not cut) + ERCP + manometry + morphine/neostigmine provocation test	Australia	NA	NA	NA	81	98%	NA	NA	NA	NA	NA	P&A	SE
van Schie et al.	2000	Diabetic foot	Silicone injection	Saline injection	UK	NA	NA	NA	28	NA	NA	NA	NA	NA	NA	P&A	LA

Note: NA – data not reported in the reviewed trial; blinded: P – patients, C – care givers, S – surgeons, A – assessors, An – analysts ; types of analgesia: GA – general anaesthesia, SE – sedation, LA – local analgesia; Condition: GERD – gastro-oesophageal reflux disease, RA – rheumatoid arthritis, GI – gastro-intestinal; Intervention: RF – radiofrequency, ERCP - endoscopic retrograde cholangiopancreatography, PET - Positron emission tomography, MRI – magnetic resonance imaging, CT – computer tomography; Countries: UK – the United Kingdom, USA – the United States of America; Sample-size: ^ - sample size was inflated to account for potential drop-out, the number given in the table is the non-inflated sample size; Blinding: refers to double- and single-blinding.
